# Fluorescence Detection of Type III Secretion Using a Glu-CyFur Reporter System in *Citrobacter rodentium*

**DOI:** 10.3390/microorganisms8121953

**Published:** 2020-12-09

**Authors:** Heather A. Pendergrass, Adam L. Johnson, Julia A. Hotinger, Aaron E. May

**Affiliations:** Department of Medicinal Chemistry, School of Pharmacy, Virginia Commonwealth University, Richmond, VA 23284, USA; pendergrassha@vcu.edu (H.A.P.); adamljohnson@outlook.com (A.L.J.); hotingerja@vcu.edu (J.A.H.)

**Keywords:** EPEC, EHEC, *C. rodentium*, T3SS, pathogenesis, natural products, CPG2

## Abstract

Enteropathogenic *Escherichia coli* (EPEC) is a major cause of infantile diarrhea worldwide. EPEC and the closely related murine model of EPEC infection, *Citrobacter rodentium*, utilize a type III secretion system (T3SS) to propagate the infection. Since the T3SS is not essential for the bacteria to survive or propagate, inhibiting the virulence factor with a therapeutic would treat the infection without causing harm to commensal bacteria. Studying inhibitors of the T3SS usually requires a BSL-2 laboratory designation and eukaryotic host cells while not indicating the mechanism of inhibition. We have designed a BSL-1 assay using the murine model *C. rodentium* that does not require mammalian cell culture. This CPG2-reporter assay allows for more rapid analysis of secretion efficiency than Western blotting and is sensitive enough to differentiate between partial and total inhibition of the T3SS. Here we present our method and the results of a small collection of compounds we have screened, including known T3SS inhibitors EGCG, regacin, and aurodox and related quorum sensing inhibitors tannic acid and ellagic acid. We have further characterized EGCG as a T3SS inhibitor and established its IC_50_ of 1.8 ± 0.4 μM. We also establish tannic acid as a potent inhibitor of the T3SS with an IC_50_ of 0.65 ± 0.09 μM.

## 1. Introduction

Exploration of novel methods for combating bacterial pathogens is necessary due to the rapid and widespread development of resistance to current antibiotics [1,2,3,4,5,6]. Rather than targeting mechanisms essential for survival, which prompts selection for resistant strains, novel research strategies aim to prevent bacteria from causing infection. By targeting virulence factors directly, pathogens may show attenuated virulence. One such method is inhibition of the type III secretion system (T3SS). The T3SS is an apparatus used as a virulence factor by many Gram-negative pathogens such as enteropathogenic and enterohemorrhagic *E. coli* (EPEC and EHEC, respectively) [7,8,9], *Salmonella enterica* serovar Typhimurium [10], *Chlamydia* spp. [11], *Yersinia pestis* [12,13,14], *Vibrio* spp., *Shigella* spp., and *Pseudomonas* spp. [12,15]. T3SS knockout strains have attenuated virulence [16], which has prompted the exploration of natural products and small molecules capable of T3SS inhibition [1,17,18,19].

**Figure 1:**
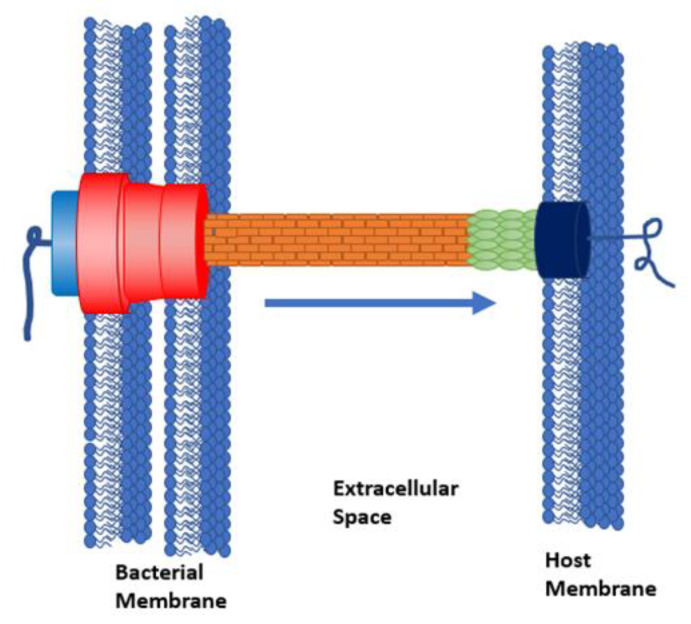
Structure of the type III secretion system (T3SS).

The T3SS functions as a molecular syringe, connecting the cytoplasm of a pathogenic cell directly to the cytoplasm of a host cell through a hollow, needle-like structure [3,20]. The pathogen then secretes virulence proteins called effectors into the host cell [21]. These injected proteins are responsible for enabling colonization and suppressing the host immune response [7,15]. The T3SS structure is composed of a basal body, a needle, and a translocon (Figure 1) [22,23]. The basal body consists of an inner and outer ring (Figure 1, red) that anchors the system into the bacterial inner and outer membranes, respectively. An ATPase (Figure 1, light blue) responsible for powering the secretion of unfolded, linear proteins is located at the base of the inner ring. The needle (Figure 1, orange) has an inner diameter of ~2.5 nm, so proteins must be linear for translocation [24,25]. Specific chaperone proteins transport unfolded effector proteins to the ATPase [26], where the proteins are recognized by a short “secretion signal” on their N-terminus [27].

Typically, assays studying the function or inhibition of the T3SS require the use of human pathogens and Biosafety Level 2 (BSL-2) conditions. These requirements may limit the ability for investigation of T3SS inhibitors for certain labs. Here we describe an assay for evaluating T3SS inhibition using the mouse pathogen *Citrobacter rodentium*. This pathogen has a BSL-1 designation and is not capable of infecting humans. *C. rodentium* encodes a T3SS with >90% sequence identity to the T3SS of EPEC and is commonly used in mouse models of EPEC infection [28]. 

Yount et al. developed an assay in *S. Typhimurium* for T3SS-mediated secretion of the enzyme carboxypeptidase G2 (CPG2) [29]. CPG2 is a protease that cleaves glutamic acid. The CPG2 gene was fused to the C-terminus of SopE2, a native effector protein, and was co-expressed with the concordant chaperone InvB. This strategy for producing a chimeric fusion of a native effector with a heterologous protein is an efficient method for secreting recombinant protein [30]. After secretion, the enzymatic activity of CPG2 was monitored as an indicator of T3SS activity [29]. We have adapted this strategy in our system to utilize information published by Munera et al., who reported N-terminal signaling sequences of effector proteins in *E. coli* that allowed for secretion [31]. The signaling sequences, heretofore referred to as “secretion signals,” were fused to the N-terminus of β-lactamase TEM-1, and the efficiency of secretion was analyzed in terms of TEM-1 activity of supernatant samples. The most efficient secretion signal reported by Munera et al. originated from effector EspF [31].

**Figure 2:**
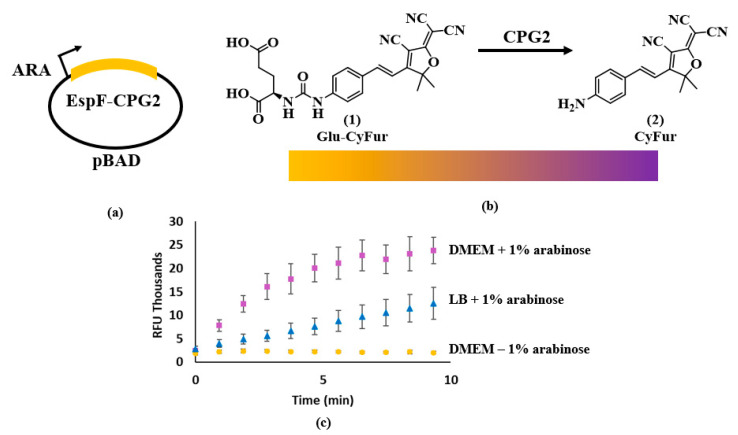
Design of the carboxypeptidase G2 (CPG2)-reporter assay. (**a**) The *cpg2* gene was tagged on the amino-terminus with the secretion signal from the T3SS effector, EspF, and expressed under an arabinose-induced promoter. (**b**) CPG2 cleaves its substrate Glu-CyFur (**1**) which releases a fluorescent compound, CyFur (**2**). (**c**) Over time, fluorescence increases as CPG2 cleave the Glu-CyFur substrate. If the T3SS is not upregulated with DMEM, the fluorescent signal is inhibited as CPG2 secretion decreases. If CPG2 is not produced (such as in the absence of arabinose), no fluorescence is produced since the Glu-CyFur substrate is not being cleaved.

Guided by the studies of Yount and Munera, we have designed and developed a CPG2 expression system in *C. rodentium* for the screening of T3SS inhibitors. The CPG2 was fused with the N-terminal secretion signal from EspF (Figure 2a). The EspF-CPG2 construct was placed in the pBAD plasmid under the control of an arabinose-induced (ARA) promoter. After secretion, CPG2 cleaved the substrate Glu-CyFur, which released fluorescent molecule CyFur (λ_ex_ = 563 nm, λ_em_ = 610 nm, Figure 2b). This cleavage also resulted in a visible color change from yellow to purple. The secretion of CPG2 was only observed in conditions that activated T3SS expression (DMEM media). When the T3SS was not active (LB media), CPG2 activity significantly decreased (Figure 2c).

**Figure 3:**
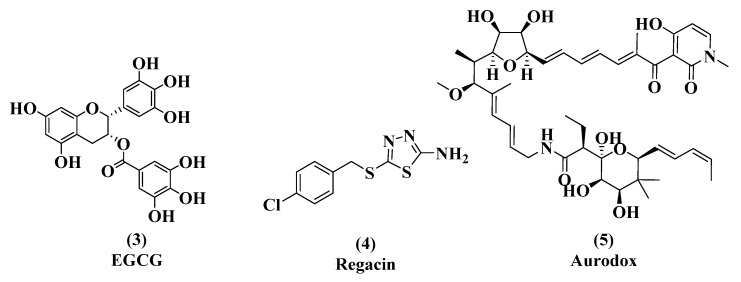
Structures of epigallocatechin-3-gallate (EGCG, (**3**), regacin (**4**), and aurodox (**5**)).

We chose to validate the CPG2 secretion assay using three T3SS inhibitors and related compounds: (-)-epigallocatechin-3-gallate (EGCG, **3**), regacin (**4**), and aurodox (**5**, Figure 3). EGCG is a polyphenolic flavonoid produced by green tea [32]. EGCG is an anti-inflammatory compound that inhibits nuclear factor-kappa B (NF-κB) activation and protein kinase C (PKC) [33]. EGCG is also being explored for its anticancer activity [34] and is a recognized inhibitor of epigenetic modifications such as DNA methylation and histone modification [35,36,37]. The versatility of EGCG has been explored extensively and it is being reviewed for its potential as a treatment option for many conditions [38]. Previous studies have indicated that at high concentrations, EGCG inhibits the T3SS of multiple organisms including EPEC [39]. Our in vitro analysis validates EGCG as a T3SS inhibitor in *C. rodentium* and indicates a concentration-dependent inhibitory profile.

Regacin reduces the expression of the *C. rodentium* T3SS by competitively inhibiting the binding of the transcriptional regulator RegA to its DNA substrates [3,40]. The interaction between RegA and DNA typically enhances type III secretion. Western blot analysis of secreted protein showed that complete inhibition of RegA-DNA interaction resulted in partial, but not complete inhibition of T3SS expression [40]. Regacin is therefore a partial inhibitor of the T3SS. We chose to study regacin to test the sensitivity of the CPG2 secretion assay to differing levels of T3SS expression.

Aurodox was originally discovered as an antibiotic for Gram-positive bacteria [41]. Recently, it has been characterized as a T3SS inhibitor in *C. rodentium* capable of attenuating infection in mice [42]. Studies on gene expression indicate that aurodox downregulates T3SS-related genes by an unknown mechanism [43]. Those same studies indicate that aurodox decreases the expression of *ler*, a major activator of the T3SS by 25% compared to untreated cells. In total, 25 of the 41 LEE-encoded genes were downregulated by treatment with aurodox. The consequence of this downregulation on the secretion of effectors is not known. However, the authors analyzed the secretion of effectors Tir and EspB/D, which are all downregulated in the presence of aurodox [43]. They also analyzed the translocation of labeled effectors into mammalian cells, which requires the pore-forming activity of EspB/D. Because our assay does not depend on translocation into a host cell and does not measure the secretion of any particular effector, we are able to distinguish between the complete abolishment of secretion and downregulation of some effectors.

**Figure 4:**
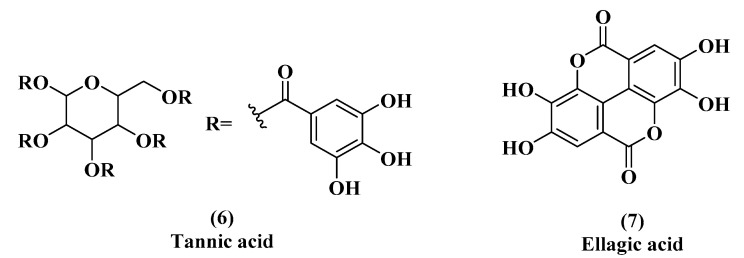
Tannic acid (**6**) and ellagic acid (**7**), quorum sensing inhibitors.

One previously published study compared the abilities of EGCG, tannic acid, and ellagic acid to inhibit quorum sensing by interfering with acyl homoserine lactone (AHL) signaling (Figure 3, EGCG & Figure 4) [44]. All three are polyphenolic compounds containing a gallic acid moiety. All three compounds indicated an ability to inhibit AHL signaling. We were interested in comparing their T3SS inhibitory activity.

## 2. Materials and Methods 

### 2.1. Materials and Compounds

Media was purchased from Difco™ (LB broth), Amresco (SOB broth), and Gibco™ (DMEM without glucose). The antibiotic chloramphenicol was purchased from Fisher. Aurodox was purchased from Santa Cruz Biotechnology, Inc. (Dallas, TX, USA). (-)-Epigallocatechin-3-gallate and nosiheptide were purchased from Cayman Chemical Company (Ann Arbor, MI, USA). Gallocatechin gallate was purchased from Alfa Aesar (Haverhill, MA, USA). Ellagic acid was purchased from Acros Organics B.V.B.A. (Fair Lawn, NJ, USA). Tannic acid was purchased from Sigma-Aldrich (St. Louis, MO, USA).

### 2.2. Cell Lines

The *cpg2* gene was amplified using polymerase chain reaction (PCR) with primers ALJ82 (5′-cgctgcttctacactagggcggcagcttgtaggtatcgcaGCTCTGGCTCAGAAACGTGA-3′) and ALJ84 (5′-TTACTTACCTGCACCCAGATCCA-3′). The pBAD vector was amplified using PCR with primers ALJ89 (5′-ATCTGGGTGCAGGTAAGTAAGTTGCTCAGGTTTCACTTGTTTTCA-3′) and ALJ88 (5′- gccctagtgtagaagcagcgTTACTAATTCCATTAAGCATGGTTAATTCCTC-CTGTTAGCCCAAA-3′) purchased from Invitrogen (Carlsbad, CA, USA). The lowercase sequences indicate the EspF secretion signal that was incorporated via PCR. Amplification was performed using HS-Taq DNA polymerase (New England BioLabs^®^, Inc., Ipswich, MA, USA). Amplified fragments were gel purified and the recombinant plasmid was assembled using the isothermal Gibson method on a T100 Thermal Cycler (Bio-Rad, Hercules, CA, USA). *C. rodentium* DBS100 (ATCC 51459) cells were made competent by washing 2× with ice-cold 10% glycerol. 70 μL-aliquots of competent *C. rodentium* cells were transformed with the pBAD-EspF-CPG2 plasmid after electroporation using a BIORAD Micropulser™. Cells were immediately resuspended in preheated SOB media and incubated at 37 °C for one hour. 5 μL, 100 μL, and concentrated pellet samples of cells were spread on LB agar plates containing 30 μg/mL chloramphenicol and incubated overnight at 37 °C. Transformants were confirmed by DNA sequencing using pBAD forward and reverse primers.

### 2.3. Enzyme Expression and Lysate Production

1 mL of an overnight culture of *C. rodentium*-EspF-CPG2 was added to 1 L LB media containing 30 μg/mL chloramphenicol and 1% arabinose (purchased from Alfa Aesar at 99% purity, resuspended in DI water and sterile filtered with a Gelman Laboratory Acrodisc^®^ (Andwin Scientific, Schaumburg, IL, USA) syringe filter, 0.2 μm) and grown overnight at 37 °C with shaking. Cells were pelleted by centrifugation at 4000 rpm for 20 min at 4 °C. Pellets were resuspended with 25 mL CPG2 buffer. Cells were lysed by sonication with a Fisherbrand™ Model 120 Sonic Dismembrator with the 1/8-inch probe. Lysate was centrifuged again to remove cellular debris before aspirating off the liquid layer.

### 2.4. Synthesis of Glu-CyFur

The Glu-CyFur substrate was synthesized as previously described [29]. This seven-step synthesis produced ~3 g of material that was used for the completion of this project. The synthesis involved the production of 4-(Boc-amino)benzaldehyde and 3-cyano-2-dicyanomethylene-4,5,5-trimethyl-2,5-dihydrofuran [45,46]. 4-(Boc-amino)benzaldehyde and 3-cyano-2-dicyanomethylene-4,5,5-trimethyl-2,5-dihydrofuran were then coupled via condensation in the presence of ammonium acetate to give (E)-*tert*-butyl-4-(2-(4-cyano-5-(dicyanomethylene)-2,2-dimethyl-2,5-dihydrofuran-3-yl)vinyl)phenylcarbamate [47]. Following acid-dissociation of the Boc protecting group, the aniline was activated for alkylation with triphosgene, then alkylated with Boc-protected glutamate to give the di-Boc-protected Glu-CyFur [29]. Deprotection Glu-CyFur. Products were confirmed by NMR or tracked by TLC.

### 2.5. Compound Screening

An overnight culture of *C. rodentium*-EspF-CPG2 (grown in LB containing 30 μg/mL chloramphenicol) was diluted 1:100 in LB containing 30 μg/mL chloramphenicol and the test compound. After three hours, the cells were pelleted by centrifugation and resuspended to an absorbance measurement at 600 nm (OD600) of 0.75 in Dulbecco’s modified Eagle’s medium (DMEM) with 1% arabinose to initiate T3SS expression and CPG2 production. The DMEM also contained the compound or vehicle control. After 6 h incubation at 37 °C, cells were pelleted and the supernatant was diluted 1:1 in buffer designed to aid in CPG2 activity (CPG2 buffer: 50 mM Tris pH 7.4, 0.1 mM ZnSO_4_, 0.04% Tween 20) in a Greiner Bio-one polypropylene black, F-shape, 384-well plate. Controls with vehicle or lacking arabinose were used. The substrate Glu-CyFur (Figure 2b) was added to each well to a final concentration of 10 µM, and fluorescence was measured with a BMG Labtech CLARIOstar© Plus plate reader (Cary, NC, USA), where λ_ex_ = 563 nm and λ_em_ = 610 nm. As glutamate is cleaved by CPG2, fluorescence increases.

### 2.6. Cytotoxicity Screening

An overnight culture of *C. rodentium* was grown in LB media at 37 °C with shaking. The culture was diluted 1:100 in LB before compounds were added at the concentrations listed and a vehicle was used as a control. 0.5-mL culture replicates of 5 were incubated for 6 h in CytoOne 96-well flat-bottom plates with the lid at 37 °C, with shaking in the BioTek Synergy HTX plate reader and incubator. Cell density was measured as OD_600_ after 6 h with the BioTek Synergy HTX plate reader and incubator (Winooski, VT, USA), beginning at the time of dilution.

### 2.7. CPG2 Direct Inhibition Analysis

These studies were conducted to ensure the compounds were not interfering with the ability of CPG2 to cleave Glu-CyFur. 1 mL of an overnight culture of *C. rodentium*-EspF-CPG2 was added to 1-L LB media containing chloramphenicol and 1% arabinose and grown overnight at 37 °C with shaking. Cells were pelleted by centrifugation at 4000 rpm for 20 min at 4 °C. Pellets were resuspended with 25 mL CPG2 buffer. Cells were lysed by sonication with a Fisherbrand™ Model 120 Sonic Dismembrator with the 1/8-inch probe. 50 μL of cell lysate containing CPG2 was transferred to a Greiner Bio-one Polypropylene black, F-shape, 384-well plate. The compound was added to the concentration listed, and a vehicle was used as a control. Glu-CyFur was added to 10 µM with a Labcyte Echo550 acoustic liquid handler, and fluorescence was observed on a BMG Labtech CLARIOstar© Plus plate reader (λ_ex_ = 563 nm and λ_em_ = 610 nm). 

### 2.8. Data Analysis

The % inhibition was calculated from the rate of change in fluorescence compared to an uninhibited control using the following equation:

$$\%\;Inhibition=100\times \left(1-\frac{m_{C}}{m_{V}}\right)$$

where *m* = slope, *C* = compound, and *V* = vehicle.

### 2.9. CPG2 Expressional Analysis

These studies were conducted to ensure the compounds screened were not interfering with the expression of CPG2 from the pBAD plasmid. We analyzed changes in CPG2 expression by comparing the rate of cleavage of Glu-CyFur of whole-cell lysate (WCL) when CPG2 expression was induced in the presence and absence of compounds. To perform these studies, the production of the CPG2 enzyme was induced in the presence and absence of the listed concentrations of compounds or an equal volume of the DMSO solvent. This was achieved by incubating the C. rodentium cells harboring the pBAD-EspF-CPG2 plasmid in DMEM with 1% arabinose and chloramphenicol. After incubating for 3 h at 37 °C without shaking. The cells were then lysed by sonication with a Fisherbrand™ Model 120 Sonic Dismembrator with the 1/8-inch probe. The lysate was then diluted 1:1 with the CPG2 buffer (CPG2 buffer: 50 mM Tris pH 7.4, 0.1 mM ZnSO4, 0.04% Tween 20) in a Greiner Bio-one polypropylene black, F-shape, 384-well plate. The substrate Glu-CyFur (Figure 2b) was added to each well to a final concentration of 10 µM, and fluorescence was measured with a BMG Labtech CLARIOstar© Plus plate reader, where λ_ex_ = 563 nm and λ_em_ = 610 nm. As glutamate is cleaved by CPG2, fluorescence increases.

## 3. Results

### 3.1. EGCG, Tannic Acid, and Ellagic Acid

**Figure 5:**
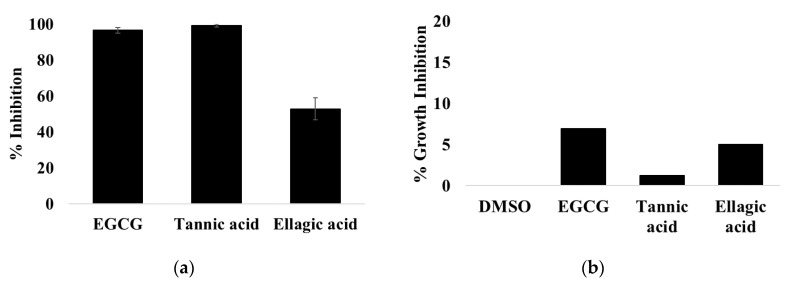
EGCG, tannic acid, and ellagic acid were screened for T3SS inhibitory activity at 50 μM. (**a**) EGCG and tannic acid appear to be more potent inhibitors than ellagic acid. (**b**) The compounds were not cytotoxic.

The three polyphenolic compounds EGCG, tannic acid, and ellagic acid, have previously been linked in their ability to inhibit quorum sensing. Here, we have analyzed their ability to inhibit the T3SS in C. rodentium. Our initial screen analyzed the ability of these compounds to inhibit the T3SS-mediated secretion of CPG2 at 50 μM (Figure 5a). The ability of these compounds to inhibit translocation of EspF-CPG2 was calculated based on the rate of Glu-CyFur cleavage by supernatant samples. At 50 μM, the compounds appear to inhibit the T3SS without affecting bacterial growth (Figure 5b).

**Figure 6:**
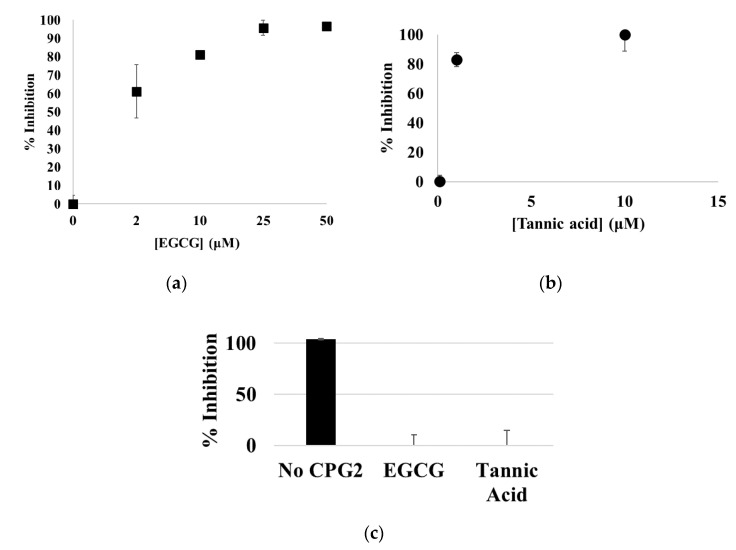
EGCG and tannic acid were analyzed at lower concentrations to determine their potency as T3SS inhibitors. (**a**) EGCG was screened at 50, 25, 10, 2, and 0 μM. The IC_50_ value of EGCG is 1.8 ± 0.4 μM. (**b**) Tannic acid was screened at 10, 1, 0.1, and 0 μM. The IC_50_ value is 0.65 ± 0.09 μM. (**c**) Neither EGCG nor tannic acid inhibits CPG2 directly at 50 μM.

EGCG and tannic acid were analyzed further to characterize their T3SS inhibitory activity. They were rescreened at lower concentrations to determine their potency. It was determined that the IC_50_ values of EGCG and tannic acid are 1.8 ± 0.4 μM and 0.65 ± 0.09 μM respectively (Figure 6). EGCG and tannic acid were then analyzed for interference in the signal. If these compounds inhibited CPG2 directly, they would appear as false positives. EGCG or tannic acid (50 μM) was added to cell lysate containing CPG2 before Glu-CyFur was added to a final concentration of 10 mM. The activity of CPG2 was then analyzed (Figure 6). The ability of these compounds to inhibit translocation of EspF-CPG2 was calculated based on the rate of Glu-CyFur cleavage by supernatant samples. It was apparent that neither EGCG nor tannic acid inhibits CPG2, indicating that they may be true inhibitors of the T3SS.

EGCG, tannic acid, and ellagic acid are structurally similar natural product quorum sensing inhibitors. Since EGCG has previously been established as a T3SS inhibitor, we characterized its inhibitory potency and determined that tannic acid and ellagic acid are inhibitors of the T3SS as well. Recent studies have shown that tannic acid was able to inhibit the T3SS-dependent host cytotoxicity prevention in *S. Typhimurium*, although the mechanism was attributed to tannic acid’s quorum sensing inhibition [48].

**Figure 7:**
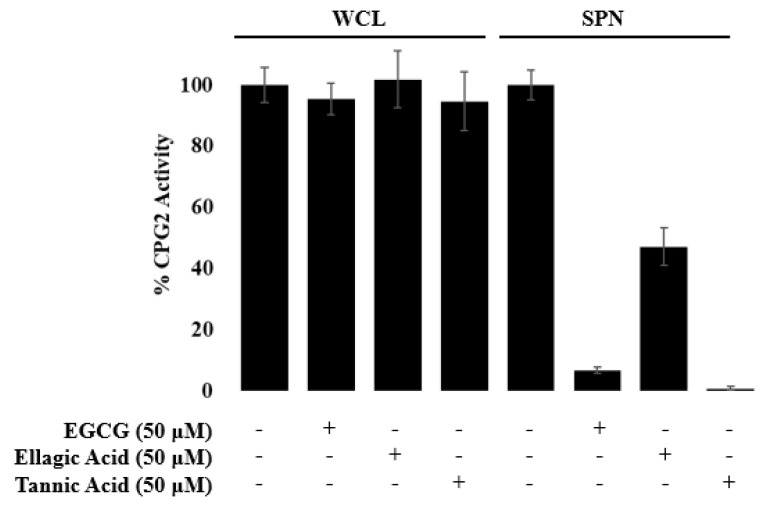
The compounds EGCG, ellagic acid, and tannic acid were screened for their ability to inhibit the production of CPG2 in whole-cell lysate (WCL) and the secretion of the enzyme into the supernatant (SPN). None of the compounds had a significant effect on the activity of CPG2 in lysate, indicating that the expression of CPG2 is not inhibited by these compounds, while still inhibiting the secretion of CPG2 through the T3SS.

To ensure these compounds are not interfering with the pBAD expression system, the CPG2 activity in whole-cell lysates were analyzed for changes to enzymatic activity in the presence of these compounds (Figure 7). Cells incubated in the presence of 1% arabinose and either test compounds or DMSO for a vehicle control. After 5 h of incubation, the cells were lysed, and the relative CPG2 concentration was analyzed as a function of Glu-CyFur cleavage. There was no significant difference in the rate of Glu-CyFur cleavage in the presence or absence of compounds, indicating these compounds have no effect on the expression of EspF-CPG2 from the pBAD plasmid. 

### 3.2. Regacin

**Figure 8:**
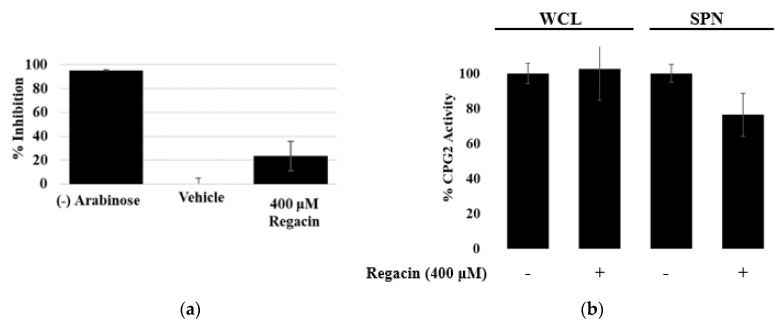
Regacin was analyzed for its ability to inhibit the T3SS-mediated secretion of CPG2. (**a**) At 40X the IC_50_ (400 μM), regacin was not able to abolish secretory activity entirely, confirming the ability of the CPG2-reporter assay to differentiate between partial and total inhibition of the T3SS. (**b**) To ensure this inhibitor of a global regulator was not inhibiting the expression of CPG2 from the plasmid, the activity of CPG2 in the presence and absence of regacin in whole-cell lysate (WCL) and supernatant (SPN) was analyzed. The activity of CPG2 in WCL was unchanged by the addition of regacin, indicating that this compound is not inhibiting the expression of CPG2. The activity of the enzyme is minorly inhibited in the SPN samples, indicating that secretion of the enzyme through the T3SS is being inhibited.

The CPG2 reporter assay is unique in that the expression of the CPG2 enzyme is independent of the expression of T3SS-related genes. This assay also does not depend upon the successful translocation of effectors into eukaryotic host cells. Because of these unique characteristics of our method, we were interested in this assay’s ability to differentiate between full and partial inhibition. Regacin is a partial inhibitor of T3SS expression capable of downregulating T3SS-related genes by competitively binding to the DNA-binding domain of a positive transcriptional regulator, RegA. When RegA is inhibited by regacin at concentrations well beyond the IC_50_, some secretion still occurs, but not enough to sustain infection in vivo. We analyzed the inhibitory activity of regacin at 400 μM, 40× the IC_50_ concentration. At that concentration, our results indicated that regacin could not completely abolish the secretion of CPG2, though the activity of the secreted enzyme was significantly inhibited (Figure 8a). This result was only possible because the expression of CPG2 was not dependent on T3SS expression. Were that the case, the concentration of CPG2 would be diminished and regacin would appear as a much more potent inhibitor of T3SS activity. One of the strengths of this assay is its ability to analyze real secretion levels as an independent parameter, much like Western blotting, but in a much shorter amount of time. Since regacin inhibits a modulator of gene expression, we analyzed whole-cell lysates for regacin’s ability to inhibit the pBAD expression system. Relative CPG2 activity was quantified by comparing the rate of Glu-CyFur cleavage by EspF-CPG2 (Figure 8b). Our results indicated that there was no change in the expression of EspF-CPG2 as a result of treatment with regacin.

### 3.3. Aurodox

**Figure 9:**
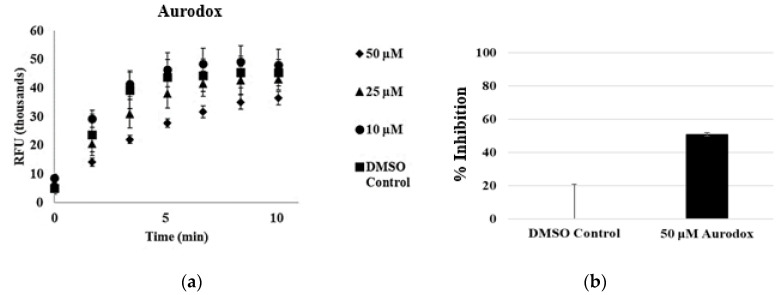
Aurodox partially inhibits type III secretion. (**a**) Increasing concentrations of aurodox results in decreases in secretion of CPG2. (**b**) High concentrations of aurodox (50 μM, 10× the IC_50_ as determined by hemolysis assays) results in ~30% inhibition of secretion.

Aurodox is a known T3SS inhibitor whose mechanism of inhibition is not fully characterized. Like regacin, it has been shown that aurodox reduced the expression of T3SS related genes. In a recent publication, expressional analysis in the presence of aurodox indicated that 25 of the 41 T3SS genes located within the locus of enterocyte effacement (LEE) were downregulated. Importantly, many of the structural components of the T3SS did not appear to be affected by aurodox. Consequently, we were interested in if secretion could still occur in the presence of aurodox. We screened aurodox at 50 μM (Figure 9) and found that secretion was partially inhibited. 

We were able to observe 32.8 ± 1.9% secretory activity at 50 μM aurodox, indicating that while aurodox inhibits the expression of effector proteins, the structural components of the T3SS may still function. The %inhibition was calculated based on the rate of Glu-CyFur cleavage from supernatant samples. This again highlights strength in the design of this assay. The in vitro analysis of aurodox inhibition has so far focused on the translocation of a reporter molecule into a eukaryotic cell. Since three of the effectors that aurodox downregulates are necessary for translocation, (EspA, EspB, and EspD) the translocation functionality of the T3SS was abolished. With the CPG2 reporter assay, we are able to better characterize the modality of inhibition and understand the global effects an inhibitor has on the virulence mechanism.

**Figure 10:**
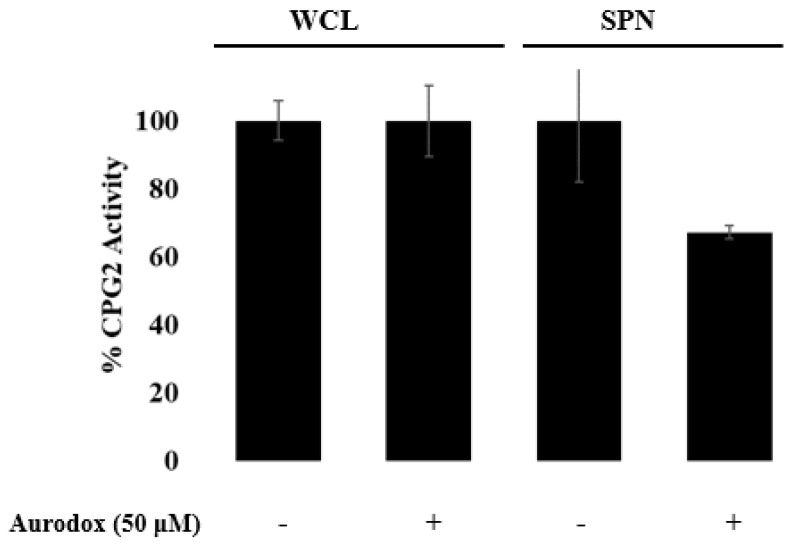
The enzymatic activity of whole cell lysate (WCL) and supernatant (SPN) samples were analyzed in the presence and absence of aurodox. Aurodox does not inhibit the expression of CPG2 in WCL, but it inhibits the secretion of CPG2 into the supernatant by the T3SS.

Given the known mechanism of aurodox as a modulator of gene expression, we analyzed whole-cell lysates for changes in EspF-CPG2 production as a result of treatment with aurodox (Figure 10). There was no apparent change in the rate of Glu-CyFur cleavage in the presence of aurodox, indicating that aurodox is not inhibiting the pBAD expression system.

## 4. Discussion

We have successfully developed an assay for monitoring T3SS activity in the presence of inhibitory compounds by modifying the published CPG2-reporter method. With our adjustments, we have further characterized the potency of the known inhibitor EGCG and other polyphenolic natural product quorum sensing inhibitors tannic acid and ellagic acid. We have determined IC_50_ values for these three compounds using the CPG2-reporter assay based on the ability of these compounds to abate secretion. 

We have indicated that this assay can differentiate between partial and total inhibition of the T3SS by analyzing the RegA inhibitor regacin. Previous Western blot analysis of regacin’s impact on the secretome has indicated that regacin partially inhibits the secretion of effectors. The CPG2-reporter assay corroborates these results and indicates that at concentrations exceeding the IC_50_, some secretion still occurs.

Guided by the understanding that aurodox downregulates the expression of secreted effector proteins without having a major impact on the structural components of the T3SS, we analyzed the activity of the *C. rodentium* T3SS in the presence of aurodox. Since the expression of CPG2 is not dependent on the expression of T3SS related genes, we were able to observe secretory activity at concentrations exceeding the previously established IC_50_ of aurodox. Our results indicate that, while aurodox successfully inhibits the translocation of effectors across the eukaryotic cell membrane, the T3SS may still be active in the presence of this inhibitor.

While this assay will be an accessible tool for the screening of new inhibitors of type III secretion, there exist weaknesses in its ability to identify all potential inhibitors. For example, regacin and aurodox are both capable of attenuating infection in vivo, though they do not inhibit the secretion of EspF-CPG2 entirely. This is due to their mechanism of downregulating, but not abolishing, T3SS protein expression. In a screening campaign for new inhibitors of the T3SS, compounds with similar mechanisms of inhibition as regacin and aurodox may show only minor inhibition. These issues notwithstanding, strong inhibition in this assay shows that the T3SS apparatus itself is not functional. This narrows down the possible mechanisms of action, and as such, this assay may be helpful in studying existing inhibitors of the T3SS.
